# Postoperative radiotherapy is effective in improving survival of patients with stage pIII-N2 non-small-cell lung Cancer after pneumonectomy

**DOI:** 10.1186/s12885-019-5692-3

**Published:** 2019-05-22

**Authors:** Wenhui Wang, Yu Men, Jianyang Wang, Zongmei Zhou, Dongfu Chen, Zefen Xiao, Qinfu Feng, Jima Lv, Jun Liang, Nan Bi, Shugeng Gao, Luhua Wang, Zhouguang Hui

**Affiliations:** 1 Department of Radiation Oncology, National Cancer Center/National Clinical Research Center for Cancer/Cancer Hospital, Chinese Academic of Medical Sciences and Peking Union Medical College, Beijing, 100021 China; 2 Department of Radiation Oncology, Peking Union Medical College Hospital, Chinese Academic of Medical Sciences and Peking Union Medical College, Beijing, 100730 China; 3 Department of VIP Medical Services, National Cancer Center/ National Clinical Research Center for Cancer/Cancer Hospital, Chinese Academic of Medical Sciences and Peking Union Medical College, Panjiayuan Nanli #17, Chaoyang District, Beijing, 100021 China; 4 Department of Thoracic Surgery, National Cancer Center/ National Clinical Research Center for Cancer/Cancer Hospital, Chinese Academic of Medical Sciences and Peking Union Medical College, Beijing, 100021 China

**Keywords:** Non-small-cell lung cancer, Stage pIII-N2, Pneumonectomy, Postoperative radiotherapy

## Abstract

**Background:**

There were few reports of postoperative radiotherapy (PORT) in stage pIII-N2 Non-small-cell Lung Cancer (NSCLC) patients receiving pneumonectomy followed by adjuvant chemotherapy. This study aims to evaluate safety and efficacy of PORT among these patients.

**Methods:**

Between Jan. 2004 and Dec. 2015, stage pIII-N2 NSCLC patients receiving pneumonectomy and adjuvant chemotherapy with or without PORT in our institution were retrospectively reviewed.

**Results:**

Totally 119 patients were included, 32 patients receiving adjuvant chemotherapy and PORT (PORT group) and 87 receiving adjuvant chemotherapy alone (Control group). There were more patients with non-R0 resection in PORT group than Control group (25% vs. 8%, *p* = 0.031). In PORT group, ≥Grade 2 radiation-induced pneumonitis was 2/32. No severe radiation-related heart injury was observed. There was no PORT-related death. Of all patients, median follow-up time was 25 months. Median overall survival time (mOS) and median disease-free survival time (mDFS) were 46 months and 15 months, respectively. The PORT group had significantly better OS (not reached vs. 34 months, *p* = 0.003), DFS (19 months vs. 13 months, *p* = 0.024), local recurrence free survival (LRFS, *p* = 0.012), and distant metastasis free survival (DMFS, *p* = 0.047) than the Control group. As for failure pattern, PORT significantly reduced local regional failure rate (39.1% vs. 15.6%, *p* = 0.016). In subgroup analysis, patients with R0 resection (*n* = 104), OS and LRFS in PORT group were significantly longer, and PORT tended to increase DFS and DMFS.

**Conclusion:**

For patients with stage pIII-N2 NSCLC after pneumonectomy and adjuvant chemotherapy, PORT can improve OS, DFS, LRFS and DMFS with tolerable toxicity.

## Background

For patients with stage pIII-N2 non-small-cell lung cancer (NSCLC) after surgery followed by adjuvant chemotherapy, postoperative radiotherapy (PORT) is a major treatment modality. Recently, a large number of retrospective studies have confirmed that 5-year overall survival (OS) could be improved by 5–6% with the application of modern radiation techniques such as three-dimensional conformal radiotherapy (3D-CRT) and intensity modulated radiation therapy (IMRT) [1–4]. However, most patients in these studies received lobectomy. At present, pneumonectomy is still an important therapeutic option for central, giant mass or tumors involving main bronchus or large blood vessels [5–7], which may bring benefits to survival [8]. As respiratory and circulatory system load (such as right ventricle) increases after pneumonectomy [5, 9], tolerance to radiation-induced pneumonitis, esophagitis and cardiac toxicity decreases after radiotherapy. Therefore, for patients with stage pIII-N2 NSCLC after pneumonectomy and adjuvant chemotherapy, safety and efficacy of PORT needs to be evaluated. The study aimed to evaluate the safety and efficacy of PORT for patients with stage pIII-N2 NSCLC after pneumonectomy and adjuvant chemotherapy.

## Methods

### Patients

Between Jan. 2004 and Dec. 2015, patients with stage pIII-N2 NSCLC who underwent pneumonectomy followed by adjuvant chemotherapy with or without PORT were retrospectively analyzed. Clinical features included: age, performance status, sex, smoking index, lung function, etc. Smoking index was defined as the number of cigarettes smoked per day plus years of smoking; preoperative pulmonary function was assessed by the absolute value of FEV1 and the percentage of FVC. Pathological information included: site of primary tumor, pathological type, tumor size, pathological stage, invasion of large vessels, lymphatic vascular invasion (LVSI), number of (positive) lymph nodes dissected and margin status. Invasion of large vessels was defined as the invasion of aorta, trunk of the pulmonary artery, superior and inferior vena cava, and any other intra-pericardial vessels.

### Treatment approaches

All patients received pneumonectomy and mediastinal lymph node dissection. Adjuvant chemotherapy consisted of 4 cycles of platinum-based doublet chemotherapy. PORT was performed according to resection status, KPS and patients’ wills with 6MV x-ray linear accelerator using IMRT or 3D-CRT techniques. Clinical target volume (CTV) included bronchial stump, ipsilateral hilum, mediastinum with or without supraclavicular area. Radiation dose was 50–60 Gy given by 1.8–2.0 Gy per fraction. Percentage of contralateral lung volume receiving at least 20Gy (V20) was restricted as equal to or less than 10% and mean dose of the contralateral lung was limited as equal to or less than 8Gy.

### Toxicity and outcome assessment

Radiation toxicities were evaluated by Common Terminology Criteria for Adverse Events Version 4.0(CTCAE). Survival durations were defined as the time from surgery to the date of death due to any cause (overall survival, OS), to treatment failure or death due to any cause (disease free survival, DFS); to locoregional failure or death due to any cause (local recurrence free survival, LRFS) or to distant metastasis or death due to any cause (distant metastasis free survival, DMFS).

### Data analysis

Data analysis was performed by SPSS statistical software (version 20.0; SPSS Inc., Chicago, IL). Chi-square test was used for categorical variables. Normality test for continuous variables was performed with Kolmogorov-Smirnov method. Student-*t* test was used to assess normal distribution variables, and Mann-Whitney U test was used for non-normal distribution variables. Kaplan-Meier method was performed to calculate survival data, and differences between groups were determined by log-rank test. A *p*-value of < 0.05 was considered as statistical significance.

## Results

### Patients and tumor characteristics

A total of 119 patients were enrolled. Characteristics were presented in Table 1. All patients had a Karnofsky performance score over 80. The median age was 53 years (range, 27 to 73 years), and 84% (100/119) were male. 78.2% (93/119) underwent left lung resection, and squamous cell carcinoma (74/119, 62.2%) was the main histologic type. According to AJCC 7th Staging System, most patients were in pathological stage IIIA (104/119, 87.4%). All patients received pneumonectomy and mediastinal lymph node dissection. R0-, R1- and R2-resection were 104/119 (87.4%), 9/119 (7.6%) and 6/119 (5%), respectively.

**Table 1:** Baseline Clinical Characteristics for Patients Treated with and without PORT

	Patients (*N* = 119)						
	Total (*n* = 119)		PORT (*n* = 32)		Control (*n* = 87)		*p*-value
Clinical Characteristic	No.	%	No.	%	No.	%	
Age, years							0.780
Median	53.0		53.5		52		
range	27–73		36–64		27–73		
Gender							0.364
Male	100	84.0	29	90.6	71	81.6	
Female	19	16.0	3	9.4	16	18.4	
Stage							0.740
IIIA	104	87.4	29	90.6	75	86.2	
IIIB	15	12.6	3	9.4	12	13.8	
Smoking index	540		600		450		0.127
Laterality							0.319
Left	93	78.2	27	84.4	66	75.9	
Right	26	21.8	5	15.6	21	24.1	
Pathology type							0.370
SCC	74	62.2	22	68.8	52	59.8	
non-SCC	45	37.8	10	31.2	35	40.2	
Pulmonary function before surgery							
FEV1(L)	2.36		2.5		2.28		0.112
FVC%	79.0		82.15		78.6		0.345
Pathologic tumor size, cm							0.281
median	5.0		4.5		5.0		
range	1.5–15.0		2.2–9.6		1.5–15.0		
Large vascular invasion	17	14.3	4	12.5	13	14.9	0.966
LVSI	29	24.4	10	31.3	19	21.8	0.289
Nerve invasion	17	14.3	5	15.6	12	13.8	0.800
LN sampled							0.867
median	25.0		25.0		26.0		
range	9–61		9–61		9–53		
Positive LN sampled							0.115
median	6.0		7.5		6.0		
range	1–33		2–33		1–25		
Resection							0.031
R0	104	87.4	24	75.0	80	92.0	
Non-R0	15	13.6	8	25.0	7	8.0	

Abbreviation: *PORT* postoperative radiotherapy, *SCC* squamous cell carcinoma, *LVSI* lymph vascular space invasion, *LN* lymph node

After pneumonectomy, 32 patients (26.9%) received adjuvant chemotherapy and radiotherapy (PORT group), while 87 patients (73.1%) underwent adjuvant chemotherapy alone (Control group). Among 32 patients in PORT group, 27 received chemotherapy followed by PORT. The other 5 received PORT followed by chemotherapy. Baseline characteristics between the two groups were comparable (*p* > 0.05), except that the rate of non-R0 resection (R1 and R2) was significantly higher in PORT group than in Control group (25% vs. 8%, *p* = 0.031). In PORT group, 25 patients (78.1%) had IMRT and 7 patients (21.9%) had 3D-CRT. The median prescription dose was 54 Gy (range, 30 to 66Gy), and median percentage of contralateral lung volume receiving at least 20Gy (V20) was 4.75% (range, 1–11.5%).

### Toxicities of radiotherapy

93.75% (30/32) patients completed PORT (completing dose above 50Gy) after pneumonectomy. One patient terminated radiotherapy at 30Gy due to chest pain, and the other patient terminated radiotherapy at 40Gy due to brain metastases. Grade 2 and grade 3 radiation pneumonitis was observed in one patient each, and the V20 of the two patients was 8.86 and 7%, respectively. There was no grade 4/5 radiation pneumonitis in PORT group. Grade 2 radiation esophagitis was observed in 10 of 32 (31.3%) patients, and no ≥ grade 3 radiation esophagitis occurred. No patients suffered significant heart damage during follow-up.

### Survival

For all patients, the median follow-up time was 25 months (range, 2–160 months). The median survival time (MST) was 46 months, and the 1-, 3- and 5-year OS were 87, 53.8 and 46.3%, respectively. The median DFS was 15 months, and the 1-, 3- and 5-year DFS were 55, 28.5 and 24.5%, respectively. Besides, the median LRFS and DMFS were 25 months and 19 months, respectively (Table 2).

**Table 2:** Impact of Different Treatment Models on Survival

	Total (*n* = 119)			R0 resection (*n* = 104)			non-R0 resection (*n* = 15)		
	PORT	Control	*p*-value	PORT	Control	*p*-value	PORT	Control	*p*-value
Case number	32	87	/	24	80	/	8	7	/
MST (mos)	NR	34	0.003	NR	34	0.032	NR	24	0.021
mDFS (mos)	19	13	0.024	18	13	0.111	33	9	0.048
mLRFS (mos)	95	22	0.012	95	22	0.033	33	9	0.045
mDMFS (mos)	47	17	0.047	19	18	0.179	112	11	0.087

Abbreviation: *PORT* postoperative radiotherapy, *MST* median survival time, *mDFS* median disease free survival, *mLRFS* median local recurrence free survival, *mDMFS* median distant metastasis free survival, *mos* months

**Fig. 1:**
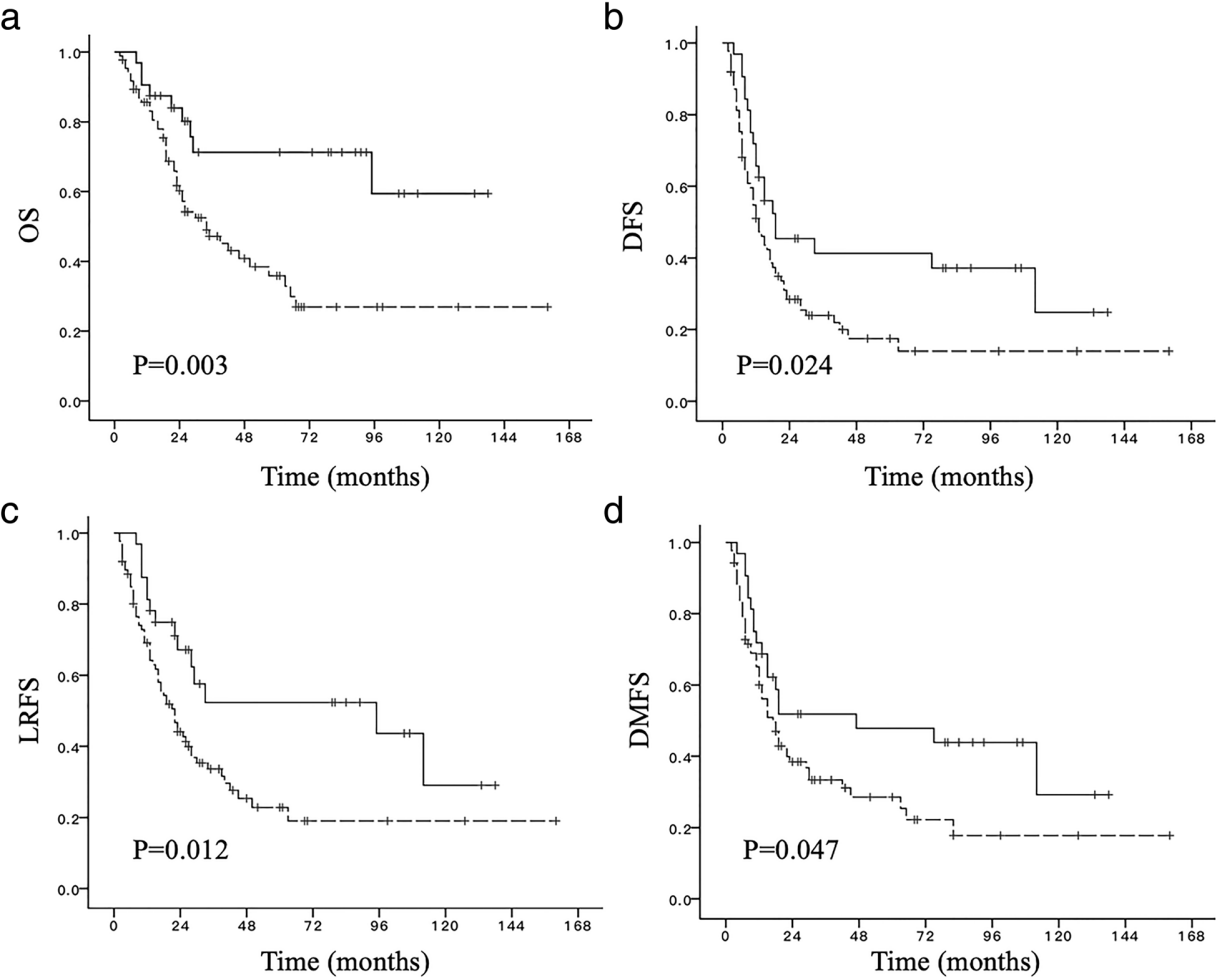
Effect of postoperative radiotherapy (PORT) on survival for stage pIIIA-N2 non-small cell lung cancer after pneumonectomy. (**a**): OS; (**b**) DFS; (**c**) LRFS; (**d**) DMFS.  PORT group;  Control group. Abbreviations: OS, overall survival; DFS, disease free survival; LRFS, local recurrence free survival; DMFS, distant metastasis free survival

The median OS in PORT group was significantly improved compared with Control group (Fig. 1a), which was not reached and 34 months, respectively (*p* = 0.003). The Fig. 1b shows the DFS curves for the both groups. The median DFS were 19 months in PORT group and 13 months in Control group, respectively (*p* = 0.024). There were significant differences in LRFS and DMFS between PORT group and Control group as shown in Fig. 1c and d. The median LRFS were 95 months and 22 months (*p* = 0.012), respectively. The median DMFS were 47 months and 17 months (*p* = 0.047), respectively. In subgroup analysis, statistically improved OS and LRFS were also found between the two groups in patients with R0 resection. The median OS was not reached versus 34 months (*p* = 0.032) and the median LRFS was 95 months versus 22 months (*p* = 0.033), respectively. PORT tended to improve DFS (*p* = 0.111) and DMFS (*p* = 0.179), with no crossing of the curves. For patients with non-R0 resection (8 in PORT group, and 7 in Control group), the OS, DFS and LRFS in PORT group was also significantly improved. While there was no significant difference of DMFS between the two groups (*p* = 0.087).

### Failure pattern

Eighty-two relapses (68.9%) were found. Of all the relapses, 58 (48.7%) were distant metastasis, and 39 (32.8%) were local-regional recurrence. Distant metastasis was the main failure pattern in both PORT group and Control group (50.0% vs. 48.3%, *p* = 0.867). However, failure occurred in local-regional site in PORT group was significantly lower than in Control group (15.6% vs. 39.1%, *p* = 0.016) (Table 3). Of the 5 patients with local-regional failure in PORT group, 4 were out of radiation field, and 1 (disrupted at 30Gy because of chest pain) within radiation field.

**Table 3:** Failure Pattern for Patients Treated with and without PORT

	Patients (*N* = 119)			
	Total (*n* = 119)	PORT (*n* = 32)	Control (*n* = 87)	*p*-value
Failure mode	No. (%)	No. (%)	No. (%)	
Local	11 (9.2)	0 (0.0)	11 (12.6)	0.035
Regional	33 (27.7)	5 (15.6)	28 (32.2)	0.074
Local and Regional	39 (32.8)	5 (15.6)	34 (39.1)	0.016
Distant	58 (48.7)	16 (50.0)	42 (48.3)	0.867

Abbreviation: *PORT* postoperative radiotherapy

## Discussion

For stage pIII-N2 NSCLC after pneumonectomy followed by adjuvant chemotherapy, the results showed good compliance and safety of PORT without any severe radiation pneumonitis and esophagitis. Compared with the control, PORT significantly improved OS, DFS, LRFS and DMFS, although there were more patients with R1/R2 resection in PORT group. To our knowledge, this is the first study focusing on the safety and efficacy of PORT for stage pIII-N2 NSCLC after pneumonectomy followed by chemotherapy.

Safety of radiotherapy after pneumonectomy is the most important concern in clinical practice. Pneumonectomy leads to great change on cardiopulmonary function. The removal of an entire lung decreases the tolerance to radiotherapy and amplifies potential risks, leading to few application of radiotherapy. In our study, 32 patients (26.9%) received PORT. In 104 patients with R0 resection, 24 cases (23.1%) were in the PORT group, which was consistent with previous reports (less than 25%) [5, 10, 11], and was significantly less than patients receiving PORT after lobectomy (43.4%) [12]. The postoperative residual tumor in gross or positive margin in microscopy was an important indication of PORT. In this study, although more patients (8/15, 53.3%) with non-R0 resection received PORT comparing with those with R0 resection (23.1%), there were still nearly half of the patients not receiving PORT, which revealed the concern on safety of PORT in both physicians and patients.

Contrary to concerns on the risks of PORT after pneumonectomy, this study showed that PORT was well tolerant with high compliance (completion rate of 93.8%). Only one patient (3.1%) suffered grade 2 radiation pneumonitis and one patient (3.1%) suffered grade 3 radiation pneumonitis, while no severe radiation pneumonitis (≥ grade 4) occurred. The incidence of ≥ grade 2 radiation pneumonitis in this study was significantly lower compared with the toxicities in previous studies of radical chemoradiotherapy (10–35%) [13–16], and previous studies of radiotherapy or chemoradiotherapy after lobectomy [17, 18]. Bradly [17] reported ≥grade 3 radiation pneumonitis with the incidence rate of 6% in 88 patients receiving adjuvant chemotherapy and concurrent radiotherapy after surgery, while pneumonectomy accounted for 14%. Besides, Zhao [18] demonstrated that the incidence of ≥grade 2 radiation pneumonitis was 13% after lobectomy, and 0% after pneumonectomy, which verified the safety of radiotherapy after pneumonectomy. Zhao believed that strict lung dose constraint and experienced beam arrangement (outside pulmonary parenchyma) were all protective and beneficial factors, which might be the reason for no serious radiation pneumonitis after pneumonectomy. In addition, in our research, low prescription dose (median dose 54Gy) and modern techniques could effectively reduce the volume and dose of the contralateral lung (median V20 4.75%), which was also an important reason for the low occurrence of radiation pneumonitis. Radiation esophagitis was another common side effect of radiotherapy. However, large-scaled analysis showed that the rate of ≥grade 3 radiation esophagitis was only 4% in NSCLC patients after postoperative chemotherapy and radiotherapy [19]. Although there were nearly 1/3 of patients suffering grade 2 radiation esophagitis in our study, no severe radiation esophagitis was noted, which may be related to the precise techniques and careful protection of normal tissue in radiotherapy after pneumonectomy. Therefore, with careful assessment of pulmonary function, reasonable prescription dose, contralateral lung dose limitation and beam arrangement, radiotherapy after pneumonectomy was safe and feasible for stage pIII-N2 patients.

In recent years, a number of retrospective studies have shown survival benefits brought by PORT in stage pIII-N2 NSCLC patients after surgery. However, most patients in these studies received lobectomy. As reported, only a few patients received pneumonectomy with the rate of 16% in Corso’s study [1], 8.3% in Robinson’s study [4], 10.8% in Hui’s study [12], 27.4% in Shen’s study [20], and highest in the ANITA study, which was only 36.9% [21]. Therefore, the benefit of PORT to survival in patients receiving pneumonectomy is not clear and still needs to be evaluated. Our study showed that PORT significantly improved OS, DFS, LRFS and DMFS in stage pIII-N2 NSCLC patients receiving pneumonectomy and adjuvant chemotherapy, although there were more patients with R1/R2 resection in PORT group. In subgroup analysis of patients with R0 resection, PORT was significantly related to better OS and LRFS, and DFS and DMFS in the PORT group trended to be improved. Even in patients with R1/R2 resection, there was a positive correlation between OS and PORT.

Local-regional recurrence is common in stage pIII-N2 NSCLC patients receiving surgery alone [22]. PORT could increase local-regional control. However, the important premise of transforming improved local-regional control into better OS was the effective prevention of distant metastasis by adjuvant chemotherapy. Thus, we enrolled patients with adjuvant chemotherapy. A meta-analysis showed that some prospective studies failed to show efficacy of PORT in improving overall survival without adjuvant chemotherapy [23]. On the other hand, a large sample retrospective analysis from national cancer database in the United States showed that the implementation of PORT on the basis of adjuvant chemotherapy, could prolong patients’ median OS by 5 months, and could increase the absolute 5-year survival rate by 5% (39.3% vs 34.8%, *p* = 0.014, 4]. Additionally, less side effects of chemotherapy and radiotherapy in patients with both R0 resection and non-R0 resection had positive impact on survival. Previous randomized trials showed that ≥grade 3 adverse events occurred in 91% of patients with postoperative concurrent chemoradiotherapy [24], which was significantly higher than that of patients treated with chemotherapy and radiotherapy. A recent retrospective study revealed that the OS in patients, with R0, R1 or R2 resection, receiving chemotherapy and radiotherapy was higher than those receiving concurrent chemoradiotherapy, although the survival benefits were not statistically significant in patients with R1/R2 resection [19]. The researchers believed that toxicities of concurrent chemoradiotherapy counteract the benefits. Therefore, pneumonectomy followed by chemotherapy and radiotherapy was a suitable mode of treatment for stage pIII-N2 NSCLC patients in order to ensure safety of surgery and adjuvant treatment.

As for failure mode, this study showed that distant metastasis was still the main reason (48.7%) of failure after pneumonectomy, followed by local-regional recurrence (32.8%). The result was consistent with the failure patterns in previously published studies, which showed that distant metastasis being the main cause of failure in stage pIII-N2 NSCLC after surgery (about 37–51%), and local-regional recurrence rate accounting for 10–35% [21, 25–27]. In several case-controlled studies enrolling stage pIII-N2 patients, PORT significantly reduced local and regional recurrence rates, compared with non-radiotherapy groups. In the ANITA study [21], local-regional recurrence occurred in 25.7% of patients, which was significantly reduced to 14.6% after combined radiotherapy. In the previous study in our center [27], 30.2% of locoregional recurrence occurred in the PORT group while 39.2% in patients without PORT (*p* = 0.025). In this study, for patients receiving pneumonectomy, PORT also achieved a significant reduction in local-regional failure compared with patients received adjuvant chemotherapy alone (15.6% vs. 39.1%, *p* = 0.016). Moreover, PORT could effectively reduce local-regional recurrence in patients after R0 resection or non-R0 resection, although the difference was not statistically significant (57.1%vs. 37.5%, *p* = 0.405) in those with non-R0 resection as small sample size (15 cases). In addition, local-regional recurrence in PORT group mostly occurred out of the radiation field (in-filed 1/5 vs. out-of filed 4/5), which also suggested the effective local-regional control of PORT. In summary, distant control still needed to be highlighted for stage pIII-N2 patients as NSCLC is a highly malignant tumor. On condition of effective prevention of chemotherapy in distant metastasis, radiotherapy could reduce local-regional recurrence and further improve survival.

Lobectomy combining with adjuvant chemotherapy and radiotherapy had showed median OS of 20–48 months, and 5-year OS of 25–61.3% for stage pIII-N2 NSCLC [3, 21, 26–29]. In the INT0139 study, stage pIII-N2 patients receiving chemotherapy and radiotherapy after pneumonectomy approached the median OS of 18.9 months, and the 5- year OS of 21.9% [25], while 5- year OS was usually lower than 20% in other studies [11, 27, 30, 31]. In this study, the median OS was 46 months and 5- year OS was 46.3%, which was similar to the prognosis of patients after lobectomy and significantly better than patients after pneumonectomy. The reasons for better prognosis in this study were as follows. Firstly, patients who died during peri-operative period were excluded. In previously reported studies, the mortality rate was high after pneumonectomy, which was about 6–20% within 30 days, and up to 25% within 6 months in some studies [5, 9]. In the INT0139 study, 14/54 died of postoperative complications after pneumonectomy [25]. Secondly, only patients receiving adjuvant chemotherapy after pneumonectomy were enrolled in this study. Thirdly, in this study, KPS scores in all patients were more than 80, and the preoperative pulmonary function approached a median FEV1 of 2.36 L. Assessment of the cardiopulmonary function was necessary before pneumonectomy. Some studies have demonstrated that patients with at least 2 L of preoperative FEV1 could tolerate pneumonectomy [7].

There were some limitations in this study. Firstly, as a retrospective, single-center based study, there might be selective bias in enrollment. Secondly, radiation induced heart injury was evaluated by heart related symptoms instead of routine ECG and echocardiography, which might lead to underestimating side effects of the heart. Thirdly, in this article, only patients who could tolerate pneumonectomy and some cycles of chemotherapy were included, which might underestimate the potential harm of pneumonectomy. However, we aimed to focus to this group of people with high KPS score and survived chemotherapy. Finally, although the clinical characteristics between PORT group and Control group were comparable except that non-R0 resection rate was higher in the former, the number of patients in the PORT group was much lower than the control group (32 patients vs. 87 patients), which might greatly weaken our findings and conclusions. Despite these limitations, this is the largest population based study exploring safety and efficacy of PORT in stage pIII-N2 NSCLC patients after pneumonectomy and adjuvant chemotherapy, which can provide high reference and guidance for PORT of stage pIII-N2 NSCLC after pneumonectomy.

## Conclusion

For patients with stage pIII-N2 NSCLC after pneumonectomy followed by chemotherapy, PORT can improve OS, DFS, LRFS and DMFS with tolerable toxicity, which makes PORT be a preferred adjuvant treatment option. Further validation is expected in the future.

## Abbreviations

3D-CRT: Three-dimensional conformal radiotherapy
CTCAE 4.0: Common Terminology Criteria for Adverse Events Version 4.0
CTV: Clinical target volume
DFS: Disease free survival
DMFS: Distant metastasis free survival
IMRT: Intensity modulated radiation therapy
LRFS: Local recurrence free survival
LVSI: Lymphatic vascular invasion
mDFS: Median disease-free survival time
mOS: Median overall survival time
MST: median survival time
NSCLC: Non-small-cell lung cancer
OS: Overall survival
PORT: Postoperative radiotherapy
